# Effects of Human Capital on Energy Consumption: The Role of Income Inequality

**DOI:** 10.3390/ijerph192417005

**Published:** 2022-12-18

**Authors:** Yiping Gao, Rong Yuan, Shenglin Zheng

**Affiliations:** School of Economics and Business Management, Chongqing University, Shazhengjie 174, Chongqing 400040, China

**Keywords:** human capital, energy consumption, AMG, mediating mechanism, income inequality

## Abstract

High-quality human capital (HC) development has a strong influence on achieving a win-win target of economic growth and energy consumption mitigation based on the background of the contemporary “carbon neutrality” constraints in China. We here aim to empirically assess the effect of HC on energy consumption (EC) in 30 provinces of China from 2000 to 2019. Moreover, we broaden the literature by discussing the effect of HC in terms of impact mechanism and nonlinear relationship. Based on methods of the augmented mean group (AMG), the estimation of long-term impacts indicates that the improvement of HC significantly discourages the increase in EC. The intrinsic mechanism shows that the accumulation of HC significantly promotes the decline of EC through economic structure adjustment and technological innovation. Moreover, the threshold model indicates that income equality lifts the inhibitory impact of HC on EC. Accordingly, the development of HC should be involved in the policy preference of China’s provincial and national development strategies considering its effectiveness in stimulating the reduction of energy consumption.

## 1. Introduction

Energy is not only the crucial input factor for production activities but also the major power source of economic development [1,2]. Since China has witnessed remarkable development, its energy consumption (EC) has experienced dramatic growth over the last 40 years [1,3,4]. China’s coal consumption increased sharply by 3.77 billion tons of standard coal during 2000–2021, at an average annual growth rate of 16.02% yr^−1^ [5,6]. Moreover, although China’s coal share decreased from 87.1% in 1965 to 56.0% in 2021, coal still accounted for a dominant share in the total EC of China [7]. China’s long-term unsustainable development pattern not only threatens energy security but also results in the occurrences of severe climate change problems (e.g., extreme climate disasters in the southwestern region of China in 2022) [8,9]. China’s CO_2_ emissions from energy have surged at full speed, ranking first with a conspicuous contribution of 31.1% for total global carbon emissions in 2021 [10]. Therefore, a quintessential issue is how to decline EC in China needs to be resolved promptly [11]. Furthermore, human capital (HC) accumulation has a significant influence on realizing sustainable development by lifting energy management efficiency [12,13,14]. Thus, the improvement of HC is likely to help address the difficulties encountered in EC reduction.

The existing literature has examined the linkage between HC and EC in China. From the perspective of micro, some studies found that households with high education levels prefer to select environmental-friendly household appliances in China [15,16,17]. However, some scholars reported that the growth of income drives the increase in electricity consumption for well-educated households in China [18,19]. The other studies indicated that labor inputs are a substitution factor for energy inputs during the production process, thereby influencing the EC pattern [20,21,22]. Moreover, previous studies from the perspective of macro discovered that HC accumulation reduces EC [23]. However, they did not use a sub-national dataset to explore the regional heterogeneity regarding this topic. Among limited studies, only Salim et al. [24] investigated the HC–EC nexus for China’s 30 provinces from 1990 to 2010 and found that HC accumulation led to EC reduction.

Nevertheless, China’s HC has risen since 2010. Approval rates of tertiary education grew by 85% during 1977–2020 [25]. Additionally, the “Central and Western Higher Education Revitalization Plan” [26] and the “High School Education Popularization Plan (2017–2020)” [27] have highlighted Chinese public commitment to the education investment, which would further promote high-quality development of higher education. Moreover, the “China Education Modernization 2035 Plan” aims to set the direction for the development of the education sector from “capacity” to “quality” [28]. Thus, we cannot ignore an updated analysis of the effect of HC on EC. In addition, previous studies did not analyze the specific conduction paths that the HC affects EC. As the rich prefer to invest in education, income inequality has proved to have an important linkage with HC [29,30]. According to Schultz’s human capital theory [31], the human capital investment includes knowledge degree, job training, skills level, and health facilities. Therefore, it is not sufficient to demonstrate the relationship between income inequality and HC that is only estimated from education investment. Meanwhile, income inequality is important for energy-saving [32]. Thus, income inequality, as an important bridge connecting HC and energy consumption, can inescapably impact the relationship between the two, even causing the nonlinear linkage between HC and EC. Therefore, assessing the specific transmission channels between HC development and EC is required, especially considering the role of inequality.

Here, using the augmented mean group (AMG) and pooled mean group (PMG) estimators, the relevant theoretical models of HC and EC are constructed, which proves the long-term impact of HC on EC. Then we come up with multi-dimensional paths through which HC impacts energy consumption. Among them, economic structure, economic growth, and technological innovation are involved. In order to provide policy support for the realization of sustainable development, we effectively assess the influence of HC on EC. We make the following contributions: First, we build a theoretical analysis framework according to HC accumulation characteristics. This framework can help explore the long-run direct effect of HC development on EC as well as the indirect transmission channels from three viewpoints of scale, structural and technical impacts. Moreover, for the purpose of identifying the nonlinear linkage between HC and EC, here we creatively use income inequality as a threshold variable. This provides important empirical evidence for China’s EC reduction action plans, considering that China’s income inequality has increased significantly, which may lead to great inequality in HC.

The remaining structure of this article is as follows. Section 2 provides the literature review. Section 3 sets out the research hypotheses. Section 4 clarifies methods, variables, as well as data sources. Section 5 presents the results. Section 6 concludes and provides policy implications.

## 2. Literature Review

### 2.1. Studies on the Human Capital–Energy Consumption Nexus

Most of the current studies used education experience as an estimated indicator for HC [33,34,35]. Several studies indicated that HC improvement has a restraining effect on EC [36,37,38]. Pablo-Romero and Sánchez-Braza [39] indicated that EC decreases when energy input is replaced with HC. Yao et al. [13] analyzed the determinants of EC across developed countries during 1965–2014 and observed that HC is negatively correlated with non-renewable energy consumption. Jiang et al. [40] observed the linkage between HC and environmental protection across 14 Asian nations during 1990–2014 and found that HC enhances environmental quality. Shahbaz et al. [14], Akram et al. [12], Sehrawat [41], and Huang et al. [42] also found that HC is in favor of EC reduction. Other scholars found HC development positively correlates with EC increase [43,44,45]. Fang and Yu [46] analyzed the association between HC, EC, and income across 16 Asia Pacific countries, and the results indicated that HC causes EC to increase. Regarding China’s case, Fang and Chen [23] confirmed the existence of a cointegration relationship between economic development, investment, HC, and EC in China. Salim et al. [24] suggested that the main pathway that can help reduce EC is HC. The conclusion of the positive association is also reached by Sarkodie et al. [47] using data from China spanning 1961–2016. Generally, current studies did not reach a consensus on the effect of HC on EC. There were studies that indicated that the linkage between HC and EC is sensitive to sample selection, econometric techniques, and HC measurement [39,48,49]. Previous studies also found a nonlinear relationship between the two [50,51,52]. Using data for G20 countries during 1970–2016, Unal et al. [53] showed that the influence of HC on the environment is diverse in geography due to the difference in the social economy and social culture factors.

### 2.2. Driving Factors of Energy Consumption

Previous studies have analyzed the influence of economic growth on EC through a series of theoretical hypotheses (e.g., growth hypothesis and feedback hypothesis) [54], and a large amount of existing literature developed a wide discussion for China’s case. However, they obtained mixed conclusions due to the difference in the methods and data sources [55,56,57,58]. Existing studies revealed the effect of population on EC based on the STIRPAT model [8,59]. Previous studies also investigated the impact of financial development on EC [60]. Sadorsky [61,62] showed that financial infrastructure affects EC by promoting economic development. Best [63] showed that in developed countries, financial development facilitates the energy structure shift toward clean energy. Moreover, Luan et al. [64] proposed the relationships between economic structure optimization and energy intensity and indicated that economic structure change leads to energy intensity reduction. Cao et al. [65] highlighted that technology innovation is primary in improving EC performance. In short, many studies indicated that economic growth, population, industrial structure, financial development, and technological innovation impact EC. Therefore, herein, we regard these factors as control variables.

## 3. Conceptual Framework and Research Hypotheses

### 3.1. Influence Mechanism of Human Capital on Energy Consumption

Here, we provide the influence mechanism as to why EC is affected by HC. From the consumption perspective, households with higher education attainment levels would like to choose energy-efficient products, thus reducing EC [15,17]. From the perspective of production, HC helps companies to promote technology innovation and create new products, thus contributing to optimal resource allocation [20,21,22]. Based on current studies, HC is related indirectly to EC by various transmission channels.

First, the expansion of HC has a pivotal impact on helping China converge to the economic development of advanced nations. The relationship between HC and economic growth has been well-established by previous studies [66,67]. The prior efforts highlighted the catalytic role of HC in supporting economic growth according to the endogenous growth theory [66,67]. Manuelli and Seshadri [68], Jones [69] and Lucas [70] indicated that HC is a key factor in driving regional economic development. Furthermore, the link between economic growth and EC is well explored on the theoretical basis of the Environmental Kuznets Curve (EKC), which assumes that the HC–EC nexus is mediated by economic development. Therefore, the accumulation of HC accelerates economic prosperity, which boosts the economic activity scale for rapid expansion, thereby leading to a surge in energy consumption.

Next, HC investment promotes technological innovation [71,72,73]. Previous studies have emphasized that HC development lifts workers’ knowledge and skills, hence stimulating technological innovation [71,74,75]. Coccia [76], Roper and Hewitt-Dundas [77], and Dong et al. [78] indicated that human resources quality affects the cost of introducing advanced knowledge. Desha et al. [79] and Mehrara et al. [80] proved that HC essentially drives the transition of energy structure through spillovers of knowledge. Thus, achieving success in the innovation process is related to HC. Meanwhile, technological innovation can encourage the application of new and clean energy technologies, thereby playing a new critical role in energy efficiency improvement and further reducing EC [81,82,83]. Particularly, the technology effect has played a prominent role in energy consumption mitigation in China relative to other countries like the United States and Japan [84]. Therefore, improving HC has a profound impact on energy savings through technology innovation.

Third, HC can lead to economic structure upgrading by improving enterprises’ innovation capacities, instructing the flow of advanced ideas to high-value-added sectors, and optimizing resource allocation [85]. Using the structural growth theory, previous analyses have discussed the influence of HC on the optimization of economic structure [86]. As HC improves, the willingness to apply new technologies in the production process increases. More advanced technologies reduce the demand for energy-intensive products and promote economic structure optimization [87]. Specifically, the development of the tertiary industry is inseparable from human resources inputs [88,89,90] because the tertiary industry, which is different from traditional industries, belongs to a technology-intensive industry with the characteristics of high value-added and energy-efficient. Considering that appropriate economic structure contributes to resource-saving and efficient energy natural management [91], updating economic structure inevitably decreases EC [92,93]. Therefore, the influence of HC on EC may depend on the upgrading of the economic structure. Here, we put forward the following hypotheses:

**Hypothesis** **I.** *Improved HC reduces energy consumption.*

**Hypothesis** **II.** *The intermediary mechanisms of the HC–EC nexus are economic growth, economic structure shifts, and technological innovation.*

### 3.2. Nonlinear Impact of Human Capital on Energy Consumption

Income inequality might produce a threshold effect when investigating the impact of HC on EC. Income inequality is closely linked to HC [94], which directly affects the general development of HC [95,96]. This means that there may be an income inequality constraint, and once income equality reaches a given level, it can fully present the influence of HC on EC. Additionally, Qadri and Waheed [97] found that HC has a greater influence on promoting economic development in low-income countries. This implies various degrees of income inequality maybe affect the effects of HC on EC. Specifically, when income is distributed unreasonably, impoverished households have fewer opportunities to obtain higher education [98]. Moreover, the enhancement of public environmental awareness caused by the equal distribution of education can strikingly stimulate EC reduction [99]. In addition, a better equal income distribution is conducive to HC allocation efficiency [30], which can have an important effect on EC. On the contrary, when the income inequality level is high, HC will flow to wealthy households. The acceptance and application of new technology may be inhibited. Such issues involving the heterogeneity of income inequality bring new challenges to the HC–EC nexus, which deserves attention. Here, we put forward the third hypothesis:

**Hypothesis** **III.** *With the change in income inequality, HC has a nonlinear effect on EC.*

## 4. Methods and Data

### 4.1. Variables and Data Sources

#### 4.1.1. Independent and Dependent Variables

The dependent variable here is energy consumption (*ec*), which is expressed in tons of standard coal equivalent and from CEADs database. The independent variable is human capital (*hc*). There is currently no authoritative measurement of the HC. Most of the current literature measured HC employing traditional statistics (e.g., pupil–teacher ratio and average years of education attainment) [100,101,102]. However, these indicators only look at the importance of schooling. They omit the key role of health situation improvement and work experience in forming HC [68,103,104]. The Jorgenson–Fraumeni (J–F) approach considers multiple perspectives of HC (e.g., work experience and health condition) by employing the income expectancy in the constant price from a perspective of life cycle [105]. China Human Capital Report by Central University of Finance and Economics using the J–F approach [106] has provided an important indicator to portray HC stock. Thus, we use the real per capita human capital stock in the constant price of 2000, which is from the *China Human Capital Index Report 2020* published by the *Central University of Finance and Economics*.

#### 4.1.2. Control and Threshold Variables

Additionally, we consider economic growth (*gdp*), capital stock (*cs*), economic structure (*is*), financial development (*fd*), population (*pop*), trade liberalization (*tra*), energy intensity (*ei*), and technological progress (*rd*) as control variables for our study as these variables have been shown to be able to impact regional energy consumption obviously. Moreover, we test the nonlinear impact of HC on EC using income inequality (that is Gini coefficient) as the threshold variable; note that it is not available for provincial data on income inequality in China. Referring to Hu [98], we estimate the provincial Gini coefficient (*gini*) using the average income of the top and bottom 20% of the total population in each province as:

(1) $$gini=\frac{\bar{H}\cdot 0.2T}{\bar{Y}\cdot T}-\frac{\bar{L}\cdot 0.2T}{\bar{Y}\cdot T}$$

where $\bar{H}$ is the average income for the top 20% of the total population; $\bar{L}$ is the average income for the bottom 20% of the total population; *T* is the total population; $\bar{Y}$ is the average income for the total population. All the variables are defined in Table 1. The data on economic growth, capital stock, economic structure, financial development, population, and trade liberalization are mainly from *China Statistical Yearbook* and *Provincial Statistical Yearbook.* The technological innovation data are from *China National Intellectual Property Administration*. 

Moreover, we aim to study the impact of HC on the EC in China’s 30 provinces. China’s HC realized fast development with rapid economic growth after 2000. The available data on EC in China’s provinces is until 2019. Therefore, we use the balanced panel data of 30 provinces in China from 2000 to 2019. Moreover, we use the corresponding price index to convert the data related to price into the constant price in 2000, which can eliminate the effect of price.

### 4.2. Methods

#### 4.2.1. Benchmark Model

Our goal is to investigate the direct impact of HC on EC. According to the model settings in past papers, we use HC as an independent variable and EC as a dependent variable. Moreover, we add eight control variables to enhance the model’s internal reliability by limiting the impact of other extraneous variables. We transform all variables in the natural logarithm to reduce the presence of heteroscedasticity, such that we directly obtain the elasticity coefficients of variables. To examine the relationship between HC and EC, we first use the conventional fixed-effects model. Because our model is cointegrated based on the panel cointegration analysis, which might bring endogeneity and efficiency loss, we use the AMG method to evaluate the long-term cointegration linkage between HC and EC. Following the principles in Pesaran’s [107], the AMG estimator considers cross-sectional dependence and country-specific heterogeneity. Moreover, the method can apprehend unobservable common elements by integrating temporal dummy variables into the model. These contribute to obtaining valid results for a cross-sectional dependent panel under the Monte Carlo simulation. We propose the basic model as follows:

(2) $$lnec=\beta_{0}+\beta_{1}lnhc+\beta_{2}lngdp+\beta_{3}lncs+\beta_{4}lnis+\beta_{5}lnfd\;+\beta_{6}lnpop+\beta_{7}lntra+\beta_{8}lnei+\beta_{9}lnrd+\alpha_{i}+\mu_{t}+\varepsilon$$

In this model, *ec* is the level of EC; *hc* is the level of HC; *gdp* is the GDP per capita, reflecting economic development level; *cs* is the capital stock; *is* is the share of labor input in the tertiary industry, representing economic structure from the labor input perspective; *fd* is the ratio of total credit to GDP, representing financial development level; *pop* is total population; *tra* is the share of total import and export in GDP, reflecting trade liberalization level; *ei* is the ratio of EC to GDP, standing for energy intensity; and *rd* is the number of green technology patents granted, representing technological innovation level. The subscripts *i* and *t* in the equations refer to province and year.

#### 4.2.2. Panel Threshold Regression Model

We analyze the nonlinear linkage of HC and EC by employing the panel threshold regression model, which is specifically shown as follows:

(3) $$\;\;\;\;lnec=\beta_{0}+\beta_{1}lnhcI\left(c<\theta \right)+\beta_{2}lnhcI\left(c>\theta \right)+\beta_{3}lngdp+\beta_{4}lncs+\beta_{5}lnis\;+\beta_{6}lnfd+\beta_{7}lnpop+\beta_{8}lntra+\beta_{9}lnei++\beta_{10}lnrd+\alpha_{i}+\varepsilon_{it}$$

where *c* and θ are the threshold variable and threshold parameter, respectively. *I* (∙) is an indicator function with a value of 1 or 0. Equation (3) shows the single threshold case, and it allows the effect of HC to vary with multi-threshold values.

#### 4.2.3. Mechanism Test

We develop an intermediary effect model to investigate the transmission mechanism for the effect of HC on EC, which is shown as:

(4) $$\ln ec_{it}=\alpha_{i}+\beta_{1}lnhc_{it}+\sum^{\;}\beta_{i}X_{it}+\varepsilon_{it}\;M_{it}=\alpha_{i}+\beta_{1}lnhc_{it}+\sum^{\;}\beta_{i}X_{it}+\varepsilon_{it}\;\;\ln ec_{it}=\alpha_{i}+\beta_{1}lnhc_{it}+\beta_{2}M_{it}+\sum^{\;}\beta_{i}X_{it}+\varepsilon_{it}$$

where *ec* and *hc* are EC and HC, respectively, and *M* is an intermediary variable. We here consider three intermediary effects, which are clarified below.

*Scale effect*: economic growth. Many researchers found that economic growth can bring scale effect [108]. Thus, we use economic growth (that is, per capita GDP) as an indicator of scale effect.

*Structural effect*: economic structure. HC improvement can affect the labor share in different industries. Moreover, some articles used labor composition to measure the structural change in economic development [87]. Thus, we assess the structural effect using the share of labor input in the tertiary industry.

*Technical effect*: technological innovation. Several scholars employed the quantity of green technology patents, which is generally accepted as a proxy for technological innovation [9]. Thus, here we regard the quantity of green technology patents granted in a region as an index of technical effect.

## 5. Results

First, using the fixed-effect and AMG estimators, we examine the potential impact of HC on the EC, which is used to verify Hypothesis I. Then we conduct an impact mechanism analysis to analyze the scale and technological and structural effects of HC on the EC, which is employed to test the Hypothesis II. Finally, we study the nonlinear effect of HC on the EC using income inequality as the threshold variable, which is applied to examine Hypothesis III.

### 5.1. Preliminary Data Analysis

We first perform a cross-sectional dependence (CD) test for methodology selection, as ignoring the CD test can cause inconsistencies in the results. We use Pesaran’s CD test to perform cross-sectional correlation. As can be seen in Table S2, the CD test shows that all variables are significant at the 1% level, which means that they all have cross-sectional dependence.

We then perform a stationary panel unit root test to evaluate the integration order of the variables. Due to the existence of cross-sectional dependence, we use the cross-sectional augmented Shin unit root test (CIPS, Im et al., 2003) [109] and the augmented cross-sectional Dickey–Fuller unit root test (CADF, Pesaran, 2007) [110] to develop unit roots. The CIPS and CADF test results in Table S3 show that all variables are significant at 1% after the first-order difference. This implies that they are all stable only after the first-order difference.

Finally, we conduct panel cointegration analysis to test the presence of long-run linkage between variables. The ADF panel cointegration test proposed by Kao [111] allows for individual fixed effects and evaluates the homogeneous cointegration linkage by the pooled regression, which is available when the timescale of the panel series is small [112]; thus, we use this method. The panel cointegration analysis in Table S4 shows that the existence of four statistics is significant, which implies that there is a strong long-run linkage between HC and EC.

### 5.2. Baseline Results

With the fixed effect model, HC has a significant negative impact on EC (see column (1) in Table 2). With a 1% increase in HC, EC reduces by 0.006%. Moreover, the result of the AMG estimator is consistent with the result from the fixed effect model (see column (2) in Table 2), showing that HC negatively affects EC. However, with a 1% increase in HC, EC reduces by 0.016%. This means that the increase of HC has a larger inhibitory effect on the increase of EC in the long run. This is due to the long-term cumulative effect of human capital, which reflects the importance of long-term investment in human capital [104].

Regarding control variables, the results show that economic development positively affects EC. A 1% increase in economic development increases EC by 1.04% and 0.887% with the fixed effect and AMG methods, respectively. This is similar to the conclusion that per capita income growth is a primary driving factor for EC [113]. In addition, the result implies that population has a great driving impact on the increase of EC. According to fixed effect and AMG methods, EC increases by 0.936% and 0.707%, respectively, when the population increases by 1%. Moreover, both energy intensity and technological innovation have driving impacts on the increase of EC with the fixed effect and AMG methods. Theoretically, technological innovation has always restrained the increase of EC by improving energy efficiency and promoting energy structure transformation. However, technological innovation also may increase EC due to rebound effects, as technological progress is the main instrument to encourage economic growth and economic development may promote the increase of energy demand in the short run [114,115,116]. Capital stock and the share of labor input in the tertiary industry have adverse impacts on EC with the fixed effect method, while there is no significant long-term effect between these variables and EC with the AMG method. In addition, there is no evident relationship between HC and trade liberalization with the fixed effect and AMG methods.

### 5.3. Robustness Tests

We use Dumitrescu–Hurlin panel causality test to explore the direction of causality between variables, as the existence of reverse causality will lead to erroneous empirical conclusions. In addition, this causality test method is applicable to panel data with cross-sectional dependence heterogeneity, which can avoid providing biased results caused by cross-sectional dependence and heterogeneity. The null hypothesis of this test is that there is no causal relationship between variables. From the results of the causality test, we can see that both W-stat and Z-stat are significant at the 10% level, which means that there is bidirectional causality between independent variables and dependent variables (Table S5). In particular, EC dose Granger-cause HC, implying that EC has a feedback effect on HC. This result is consistent with the study showing that nuclear energy consumption promotes human development [117].

Moreover, we use multiple approaches to ensure the robustness of the results. First, we use the approach of removing extreme values to check the robustness (see Table S6). After excluding the effects of extreme values, we find that the AMG result of HC on EC is insignificant, while the fixed effect result is significant. Next, we use the per capita EC as the substitute variable for EC (see Table S7). The results suggest that the coefficient and significance of the independent variable have no obvious change. Moreover, the per capita average year of formal schooling is used as the substitute variable for HC (see Table S8). The results show that there is no significant change in the coefficient with fixed effect and AMG methods, but the significance with the AMG method increases from 10% to 1%. Moreover, the AMG estimator can only judge the long-term relationship. Here we employ the PMG method to investigate the long and short terms effects of HC on EC. Table S9 shows that HC has a negative effect on HC in the long term, as indicated by a coefficient of −0.271 with a 1% significance level. However, HC has no obvious impact on EC in the short term. This is because of the changing relationship between HC and EC. The HC improvement always experiences a long time of accumulation and has lagged impact on EC. Thus, in the HC investment process, its energy impact is not significant. However, from a long-term perspective, the relationship between HC and EC would change, thereby influencing their significance level. This result is consistent with the result of the AMG method that HC has a significantly negative effect on EC in the long term.

Finally, the choice of samples often impacts the estimated results. To test whether the impact of the HC on energy varies by region, we split China’s 30 provinces into coastal and inland regions and calculated the long-term impact of HC on energy consumption. We find that the impact of HC on EC presents great heterogeneity (see Table S10). Even more specifically, HC has a negative impact on EC in the coastal region, showing a coefficient of −0.054 and a significance level of 5%, while it is not significant for the coefficient of HC in the inland region. This may be due to the differences in HC development levels across the two regions. There is a large gap in the economic growth between inland and coastal regions, and previous studies have argued that the unequal distribution of economic growth can cause HC inequality among Chinese regions. This is because coastal provinces can attract large human resources due to improvements in social welfare, rapid urbanization, and the influence of economic reform during the last years. In addition, income inequality has an impact on the relationship between HC and EC based on the analysis of Section 5.5. In the inland region, the Gini coefficients in 100% of inland provinces surpass 0.26, while the Gini coefficients in 27.3% of coastal provinces are smaller than 0.26. Relatively higher income inequality in the inland region may be a reason for the insignificant energy impact of HC.

### 5.4. Mediation Effect Analysis

We examine the influence mechanism of HC on EC by analyzing the scale effect, structural effect, and technical effect. First, columns (1) and (2) of Table 3 aim to check the scale effect. In column (1), we can see the negative and significant coefficient (−0.017) of HC, which means that HC reduces EC. However, the coefficient of HC is insignificant in column (2). This indicates the HC itself seems not to be a guarantee of economic development. This is because the impact of HC on economic growth is different for all economic development stages. Sultana et al. [118] indicated that in the developed region, the economic impact of HC becomes insignificant. The main reason for the insignificant impact of HC on economic growth may be that China is gradually entering into developed countries’ economic growth model with fast development and economic structure shift. Thus, the scale effect of HC is invalid.

Second, columns (3) and (4) of Table 3 aim to check the structural effect. In column (3), the negative and significant coefficient (−0.007) of HC suggests that HC reduces EC. In column (4), HC has a positive effect on the economic structure shift, showing a coefficient of 0.057 with a 1% significance level. This is because it is easy for well-educated individuals to absorb new knowledge, leading to the introduction of advanced technologies. Such changes in the production process favor the adjustment in economic structure and specialization, leading to the growth of high-value-added industries (e.g., services) [87]. This implies the structural effect is a critical channel for linking EC and HC.

Third, columns (5) and (6) of Table 3 show that the coefficients of HC are both negative, which are all significant at 5%. This indicates that HC reduces EC, while the effect of HC on technological innovation is negative. Generally, the HC, which reflects a highly educated and high-quality workforce, improves the technological innovation levels by proposing new ideas [119]. However, protected by the country’s regulatory forces, the energy sector, which belongs to the monopolistic sector, always obtains resources at lower market prices and obtains the support of government policies. Influenced by this incentive mechanism, HC has limited interest in joining innovation activities according to market demand and price signals but looks at rent-seeking activities, which further discourages technological innovation. Moreover, affected by the economic shock, the distortion of HC causes the gap in relative remuneration of HC among sectors, such that HC prefers to flow from competitive sectors with low compensation to monopolistic sectors with high compensation. This would cause the HC mismatch, which means that HC could not be allocated effectively. Therefore, the oversupply of HC in monopolistic sectors increases rent-seeking activities, while in competitive sectors, the demand for HC surpasses its supply. These unavoidably result in a decline in technological innovation level because of the lack of a high-quality labor force for technology-intensive enterprises. Moreover, technological innovation drives the increase of EC; thus, technological innovation has a driving impact on the negative HC–EC nexus. This indicates that the technical effect of HC is valid.

### 5.5. Panel Threshold Analysis

**Figure 1:**
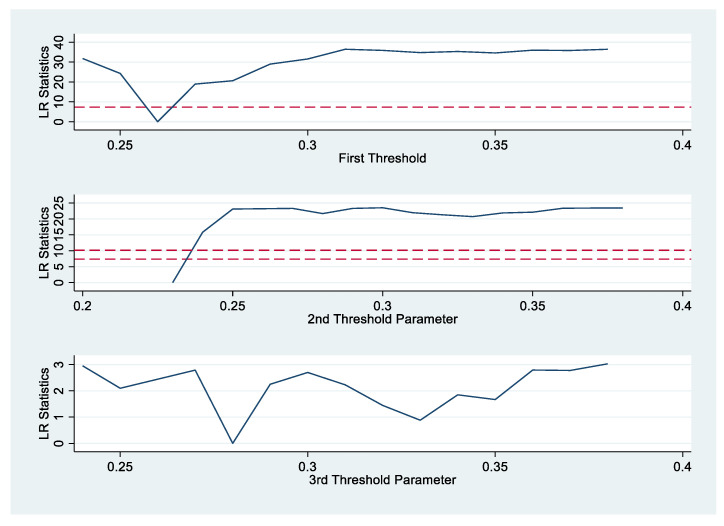
Threshold results of HC. Note: solid blue line indicates LR. Red dotted line indicates the 95% value.

Table 4 shows that the single threshold is significant at a 1% level, which implies HC has a significant nonlinear effect on EC. The results confirm that when the income inequality is smaller than the first threshold value (−0.260), the effect of HC on EC is significantly negative (see Table 5 and Figure 1). Once the income inequality exceeds the first threshold (−0.260), the coefficient of HC becomes insignificant. A higher Gini coefficient indicates larger inequality. The Gini coefficient that is smaller than 0.26 means that income is distributed quite equally. Thus, the improvement of HC could drive a reduction in the EC only when income is equally allocated. Therefore, the reduction in income inequality is an important impetus for the effect of HC on EC. The results of the control variables show that economic development, financial development, increased population, increased energy intensity, and technological innovation promoted the increase of EC, while capital stock increases and economic structure shifts decreased EC. Moreover, there is no significant effect of trade liberalization on EC.

In summary, we have confirmed Hypothesis III, which posits that HC has a nonlinear effect on EC with the change in income inequality. When the income Gini coefficient rises, the impact of HC on reducing EC decreases and become insignificant. This implies the relationship between HC and EC does not change monotonically. A significant shift in their relationship may depend on equal income distribution as a necessary condition. Thomas et al. [120] justified that HC does not have the characteristic of being tradable; thus, it is not an equalized marginal product for all people. Consequently, the total EC not only relies on the average level of HC but also is affected by its distribution. Hu [98] indicated that HC inequality increases when income inequality rises. This may be a reason why income inequality influences the impact of HC on EC.

## 6. Conclusions

### 6.1. Conclusions

We investigated the impact of HC on EC in China from 2000 to 2019 using the fixed effect, AMG, and PMG estimators. Moreover, we analyzed how HC affects EC by distinguishing three transmission channels (i.e., scale effect, structural effect, and technical effect). Primarily, we used income inequality as a threshold variable, exploring whether there is a nonlinear relationship between HC and EC. Finally, we discussed the heterogeneous impact of HC on EC between inland and coastal regions.

We found that the HC improvement reduces EC with the fixed effect, AMG, and PMG estimators. This conclusion supports previous studies that suggest HC enhances environment quality by improving management efficiency [121]. However, our result differs from the conclusions that HC works by maximizing resource utilization [121]. Regarding the underlying channels, we found that increased HC stimulates economic structure upgrading, decreasing energy consumption. High HC suppresses technological innovation, thereby reducing energy consumption. In addition, we found the nonlinear threshold role of income inequality in the interaction between HC and EC. Generally, the increase in inequality reduces the inhibitory effect of HC on the increased EC. This can be explained by the response pattern of HC investment to the increased income inequality [98]. In general, due to limited financial capacity, the poor are not encouraged to obtain a higher education level. However, the rich hope that HC can be transformed into wealth, thereby increasing investment in education. Therefore, when income inequality reaches a certain level, a large amount of HC will be amassed by a small share of elites. Because the vast majority of households with insufficient education may be related to a lower preference for environmental protection, the negative impact of HC on EC is insignificant. Finally, we found that in the coastal region, the improvement of HC has a negative impact on the increase of EC, while there is no convincing evidence for the inhibitory effect of HC on EC in the inland region.

### 6.2. Policy Implications

We have several policy implications according to our results. First, our results, which showed a negative relationship between HC and EC, suggest that in order to reduce China’s energy consumption, policymakers should foster the accumulation of HC by involving more investments in education and health systems and improving the share of higher education levels. This means that China’s climate mitigation goal is consistent with current educational strategies and policies, such as the “Education Modernization 2035 Plan”, which stipulates enhancing the quantity of HC.

Next, our results showed that income equality could strengthen the inhibitory impact of HC on EC. Thus, policymakers should smooth the income gap between poor and rich households by initiating poverty reduction programs and living standard guarantee programs. Moreover, many other dimensions of development, especially improving public services for poor households, are important. For example, the government should promote industrialization and encourage technology transfer in rural areas, considering most poor Chinese households are from rural areas. Moreover, policymakers should implement a redistribution policy to further protect the rights of health care and education for poor households. Finally, the government should adopt various financial measures to ease credit imperfections faced by poor households, especially by providing poor households with more diverse financial assistance to access education (e.g., financial assistance programs in the forms of national scholarships and tuition-cost waivers).

Third, since the improvement of inequality has no negative influence on EC in the inland region, policymakers should further optimize the allocation of HC resources between developed coastal and less developed inland regions, especially strengthening the education investment in the inland region. This means that China should make efforts to promote convergence in HC while encouraging the construction of a sustainable development economic system in the inland region. Various policies, such as reform aimed at expanding the coverage of compulsory education in rural areas [122] and the “Central and Western Higher Education Revitalization Plan” [26] have been implemented to promote the optimization of HC resources. Our result further implies that these policies, which create equal opportunities for households in less developed regions to participate in educational activities, are crucial to realize the energy reduction goal.

### 6.3. Future Studies

It is necessary to highlight that although our results are useful for investigating the relationship between HC and EC, the dataset has a limited competitive advantage because of its inaccessibility to sectoral-level or even firm-level data. We hope to further analyze the related issues (e.g., energy intensity and clean energy consumption) based on a competitively advantaged dataset. This can provide policymakers with a comprehensive interpretation of the energy impact of HC. Moreover, human capital development is involved in a systematical economic–energy analysis framework, which not only provides an interesting viewpoint for China’s energy consumption mitigation but also extends the research range in the subject of energy economics for emerging countries, considering that China is the top energy consumer. Further related studies can be applied to other emerging regions.

## Figures and Tables

**Table 1:** Definition of variables.

Variable Name	Definition
Energy consumption (*ec*)	The level of energy consumption
Human capital (*hc*)	The level of human capital
Economic development level (*gdp*)	The GDP per capita
Capital stock (*cs*)	The capital stock
Economic structure (*is*)	The share of labor input in the tertiary industry
Financial development level (*fd*)	The ratio of total credit to GDP
Population (*pop*)	The total population
Trade liberalization level (*tra*)	The share of total import and export in GDP
Energy intensity (*ei*)	The ratio of EC to GDP
Technological innovation level (*rd*)	The number of green technology patents granted
Income inequality (*gini*)	The average income of the top and bottom 20% of the total population in each province

**Table 2:** Baseline results.

Variables	FE	AMG
*lnhc*	−0.006 *	−0.016 *
	(−1.940)	(−1.840)
*lngdp*	1.049 ***	0.887 ***
	(68.492)	(46.084)
*lncs*	−0.020 ***	−0.003
	(−4.535)	(−0.579)
*lnis*	−0.016 *	−0.010
	(−1.957)	(−1.208)
*lnfd*	0.014	−0.052 ***
	(1.157)	(−5.173)
*lnpop*	0.936 ***	0.707 ***
	(43.717)	(8.048)
*lntra*	0.003	0.001
	(1.219)	(0.587)
*lnei*	0.982 ***	0.998 ***
	(150.165)	(224.460)
*lnrd*	0.014 ***	0.003 *
	(5.220)	(1.861)
Constant	−7.900 ***	−4.129 ***
	(−21.627)	(−2.724)
Obs	600	600

Note: *** indicates significance at 1%, * indicates significance at 10%.

**Table 3:** Mechanism test results.

Variables	*lnec*	*lngdp*	*lnec*	*lnis*	*lnec*	*lnrd*
*lnhc*	−0.017 **	−0.011	−0.007 **	0.057 ***	−0.007 **	−0.086 **
	(−2.116)	(−1.453)	(−2.399)	(3.760)	(−2.455)	(−2.096)
*lngdp*			1.038 ***	0.137	1.062 ***	1.653 ***
			(59.767)	(1.545)	(62.921)	(7.148)
*lncs*	0.038 **	0.077 ***	−0.042 ***	−0.023	−0.039 ***	0.306 ***
	(2.148)	(4.922)	(−6.516)	(−0.701)	(−5.915)	(3.425)
*lnis*	0.019	0.032			−0.012	0.169
	(0.811)	(1.545)			(−1.441)	(1.451)
*lnfd*	−0.336 ***	−0.299 ***	−0.025 *	0.037	−0.012	0.936 ***
	(−9.365)	(−9.317)	(−1.781)	(0.513)	(−0.859)	(4.904)
*lnpop*	−0.007	−0.860 ***	0.885 ***	0.124	0.909 ***	1.679 ***
	(−0.137)	(−17.886)	(36.041)	(0.990)	(37.310)	(5.028)
*lntra*	0.011	0.006	0.004	0.013	0.005	0.044
	(1.316)	(0.863)	(1.341)	(0.885)	(1.588)	(1.096)
*lnei*	0.809 ***	−0.175 ***	0.990 ***	0.064	0.995 ***	0.290 **
	(38.844)	(−9.375)	(121.600)	(1.546)	(120.930)	(2.575)
*lnrd*	0.067 ***	0.052 ***	0.013 ***	0.023		
	(8.305)	(7.148)	(4.222)	(1.451)		
Constant	7.664 ***	13.954 ***	−6.861 ***	1.015	−7.228 ***	−28.621 ***
	(7.604)	(15.448)	(−15.614)	(0.452)	(−16.553)	(−4.785)
Observations	600	600	600	600	600	600
R-squared	0.980	0.845	0.997	0.661	0.997	0.962

Note: *** indicates significance at 1%, ** indicates significance at 5%, * indicates significance at 10%.

**Table 4:** Threshold effect test.

Threshold	RSS	MSE	F	P	10%	5%	1%
Single	0.257	0.000	87.11	0.006	32.831	42.058	68.999
Double	0.247	0.000	24.01	0.104	24.175	28.976	37.211
Triple	0.246	0.000	3.11	0.884	20.808	25.786	42.279

**Table 5:** Panel threshold regression results.

Variables	Coefficients	Variables	Coefficients
*lngdp*	1.028 ***	*lnei*	0.975 ***
	(71.028)		(158.337)
*lncs*	−0.016 ***	*lnrd*	0.016 ***
	(−3.846)		(6.029)
*lnis*	−0.013 *	*lnhc*(*c* < 0.2600)	−0.011 ***
	(−1.694)		(−4.032)
*lnfd*	0.018 *	*lnhc*(*c* > 0.2600)	−0.004
	(1.660)		(−1.460)
*lnpop*	0.921 ***	Constant	−7.704 ***
	(45.959)		(−22.556)
*lntra*	0.004	Observations	600
	(1.423)	R-squared	0.998

Note: *** indicates significance at 1%, * indicates significance at 10%. *c* is the threshold variable.

## Data Availability

Data are available on the request.

## Supplementary Materials

The following supporting information can be downloaded at: https://www.mdpi.com/article/10.3390/ijerph192417005/s1. Table S1: Summary statistics; Table S2: CD test; Table S3: CIPS and CADF tests; Table S4. Kao co-integration test; Table S5: Results of Dumitrescu-Hurlin panel causality test; Table S6: Results of fixed effect and AMG estimators after removing extreme value; Table S7: Alternative measure of energy consumption: per capita energy consumption; Table S8: Alternative measure of human capital: Per capita average years of formal schooling; Table S9: PMG estimation results; Table S10: Estimated results of inland and eastern regions with the AMG estimator.
