# Supplementary material for: Effects of Human Capital on Energy Consumption: The Role of Income Inequality
Source: Int J Environ Res Public Health. 2022 Dec 18;19(24):17005. doi: 10.3390/ijerph192417005 (PMC9778858; doi:10.3390/ijerph192417005)
Supplement: Supplementary file 1 [file ijerph-19-17005-s001.zip › ijerph-2018328-supplementary.pdf]

SI Appendix

Table S1 Summary statistics

Variables	N	Mean	SD	Min	Max
lnec	600	9.010	0.800	6.174	10.631
lnhc	600	4.053	0.726	1.883	6.002
lngdp	600	-0.164	0.453	-1.298	1.096
lncs	600	9.573	1.131	6.605	11.94
lnis	600	3.539	0.283	2.830	4.551
lnfd	600	1.018	0.312	0.374	2.019
lnpop	600	17.37	0.754	15.46	18.56
lntra	600	2.314	2.424	-7.210	5.142
lnei	600	1.012	0.573	-0.303	2.666
lnrd	600	6.009	1.968	0.000	10.35
gini	600	0.315	0.0514	0.170	0.430

Table S2 CD test.

Variables	Statistic	p-value
lnec	88.55***	0.000
lnhc	66.73***	0.000
lngdp	54.59***	0.000
lncs	92.22***	0.000
lnis	65.92***	0.000
lnfd	77.34***	0.000
lnpop	44.47***	0.000
lntra	28.70***	0.000
lnei	73.61***	0.000
lnrd	91.11***	0.000

Note: *** indicates significant at 1%.

Table S3 CIPS and CADF tests.

Variables	CIPS		CADF	
	Original	First order difference	Original	First order difference
lnec	-1.535	-3.185***	-2.106*	-3.031***
lnhc	-0.836	-2.844***	-0.962	-3.216***
lngdp	-1.547	-2.604***	-2.266**	-2.530***
lncs	-1.970***	-1.865***	-3.309***	-2.283**
lnis	-1.752**	-2.948***	-1.410	-2.966***
lnfd	-1.829***	-2.617***	-2.079*	-2.235**
lnpop	-1.375	-2.180***	-1.591	-2.387***
lntra	-1.145	-3.392***	-0.822	-2.645***
lnei	-1.591	-2.972***	-1.933	-2.820***
lnrd	-2.007***	-3.553***	-2.063	-3.005***

Note: *** indicates significant at 1%, ** indicates significant at 5%, * indicates significant at 10%.

Table S4 Kao co-integration test

	Statistic	p-value
Modified Dickey-Fuller t	3.017	0.001
Dickey-Fuller t	3.567	0.000
Augmented Dickey-Fuller t	2.294	0.010
Unadjusted modified Dickey-Fuller t	3.438	0.000
Unadjusted Dickey-Fuller t	4.187	0.000

Table S5 Results of Dumitrescu-Hurlin panel causality test

Hypothesis	W-star	Z-star	P-value	results
lnhc→*lnec*	1.6490	2.5137**	0.0119	yes
lnec→*lnhc*	1.7435	2.8796***	0.0040	yes
lngdp→*lnec*	2.6462	6.3755***	0.0000	yes
lnec→*lngdp*	5.4362	17.1814***	0.0000	yes
lncs→*lnec*	2.0227	3.9607***	0.0001	yes
lnec→*lncs*	60.0015	228.5120***	0.0000	yes
lnis→*lnec*	2.0582	4.0983***	0.0000	yes
lnec→*lnis*	3.0127	7.7951***	0.0000	yes
lnfd→*lnec*	1.4747	1.8387*	0.0660	yes
lnec→*lnfd*	2.4266	5.5253***	0.0000	yes
lnpop→*lnec*	2.1268	4.3639***	0.0000	yes
lnec→*lnpop*	12.4279	44.2599***	0.0000	yes
lntra→*lnec*	2.6033	6.2095***	0.0000	yes
lnec→*lntra*	3.4129	9.3451***	0.0000	yes
lnei→*lnec*	2.9222	7.4445***	0.0000	yes
lnec→*lnei*	2.4883	5.7643***	0.0000	yes
lnrd→*lnec*	2.0609	4.1088***	0.0000	yes
lnec→*lnrd*	6.5379	21.4481***	0.0000	yes

Note: *** indicates significant at 1%, ** indicates significant at 5%, * indicates significant at 10%.

Table S6 Results of fixed effect and AMG estimators after removing extreme value.

Variables	FE	AMG
lnhc	-0.009***	-0.008
	(-2.679)	(-0.947)
lngdp	1.055***	0.903***
	(57.524)	(47.595)
lncs	-0.020***	-0.001
	(-3.791)	(-0.130)
lnis	-0.019**	-0.013*
	(-1.971)	(-1.648)
lnfd	0.013	-0.045***
	(1.013)	(-3.466)
lnpop	0.908***	0.697***
	(35.135)	(6.863)

lntra	0.005	-0.000
	(1.575)	(-0.084)
lnei	0.988***	1.001***
	(129.269)	(151.777)
lnrd	0.014***	0.003*
	(4.209)	(1.767)
Constant	-7.395***	-4.578**
	(-16.822)	(-2.271)
Obs	515	505

Note: *** indicates significant at 1%, ** indicates significant at 5%, * indicates significant at 10%.

Table S7 Alternative measure of energy consumption: per capita energy consumption.

Variables	FE	AMG
lnhc	-0.006*	-0.016*
	(-1.940)	(-1.842)
lngdp	1.049***	0.887***
	(68.492)	(46.084)
lncs	-0.020***	-0.003
	(-4.535)	(-0.579)
lnis	-0.016*	-0.010
	(-1.957)	(-1.209)
lnfd	0.014	-0.052***
	(1.157)	(-5.172)
lnpop	-0.064***	-0.293***
	(-2.997)	(-3.335)
lntra	0.003	0.001
	(1.219)	(0.588)
lnei	0.982***	0.998***
	(150.164)	(224.473)
lnrd	0.014***	0.003*
	(5.220)	(1.861)
Constant	-7.900***	-4.129***
	(-21.626)	(-2.724)
Obs	600	600

Note: *** indicates significant at 1%, ** indicates significant at 5%, * indicates significant at 10%.

Table S8 Alternative measure of human capital: Per capita average years of formal schooling.

Variables	FE	AMG
lneducation	-0.056*	-0.068***
	(-1.670)	(-2.587)
lngdp	1.051***	0.883***
	(68.040)	(93.473)
lncs	-0.020***	0.000
	(-4.450)	(0.056)

lnis	-0.018**	-0.011*
	(-2.303)	(-1.715)
lnfd	0.010	-0.054***
	(0.859)	(-7.893)
lnpop	0.931***	0.585***
	(43.802)	(5.509)
lntra	0.005*	0.000
	(1.663)	(0.069)
lnei	0.984***	1.002***
	(139.486)	(203.672)
lnrd	0.016***	0.003**
	(5.471)	(2.376)
Constant	-7.722***	-2.244
	(-21.039)	(-1.247)
Obs	600	600

Note: *** indicates significant at 1%, ** indicates significant at 5%, * indicates significant at 10%.

Table S9 PMG estimation results.

Varables	Long-run coeff	Short-run coeff
lnhc	-0.271***	-0.001
	(-4.336)	(-0.098)
lngdp	1.255***	1.003***
	(9.128)	(72.297)
lncs	0.359***	0.021***
	(5.876)	(2.803)
lnis	-0.334***	-0.003
	(-4.258)	(-0.375)
lnfd	-0.353***	-0.009
	(-5.153)	(-1.303)
lnpop	1.751***	0.665***
	(10.790)	(3.490)
lntra	-0.099***	0.001
	(-3.724)	(0.177)
lnei	0.849***	1.012***
	(17.999)	(121.454)
lnrd	0.012	0.002
	(0.790)	(1.268)
ec		0.012*
		(1.689)
Constant		0.262*
		(1.665)
Obs	600	600

Note: *** indicates significant at 1%, ** indicates significant at 5%, * indicates significant at 10%.

Table S10 Estimated results of inland and eastern regions with the AMG estimator.

Variables	Inland region	Coastal region
lnhc	-0.006	-0.054**
	(-0.694)	(-2.194)
lngdp	0.980***	0.829***
	(56.020)	(21.643)
lncs	0.025***	0.019
	(4.790)	(0.903)
lnis	-0.003	0.008
	(-0.484)	(0.824)
lnfd	-0.033***	-0.058***
	(-3.079)	(-2.860)
lnpop	1.045***	0.404***
	(8.231)	(2.606)
lntra	-0.001	0.006
	(-1.089)	(0.995)
lnei	1.000***	0.997***
	(173.206)	(47.347)
lnrd	0.002*	-0.002
	(1.775)	(-0.637)
Constant	-10.274***	1.387
	(-4.798)	(0.530)
Obs	380	220

Note: *** indicates significant at 1%, ** indicates significant at 5%, * indicates significant at 10%.
